# PEI/MMNs@LNA-542 nanoparticles alleviate ICU-acquired weakness through targeted autophagy inhibition and mitochondrial protection

**DOI:** 10.1515/biol-2022-0952

**Published:** 2024-09-09

**Authors:** Yun Wang, Yi Xu, Tun Zhao, Ya-Jun Ma, Wei Qin, Wen-Li Hu

**Affiliations:** Department of Neurology, Beijing Chaoyang Hospital, Capital Medical University, Beijing 100020, China; Department of Pharmacy, First Affiliated Hospital of Wenzhou Medical University, Wenzhou, Zhejiang, 325000, China

**Keywords:** intensive care unit- acquired weakness, MiR-542, ATG5, cellular autophagy, mitochondrial damage

## Abstract

Intensive care unit-acquired weakness (ICU-AW) is prevalent in critical care, with limited treatment options. Certain microRNAs, like miR-542, are highly expressed in ICU-AW patients. This study investigates the regulatory role and mechanisms of miR-542 in ICU-AW and explores the clinical potential of miR-542 inhibitors. ICU-AW models were established in C57BL/6 mice through cecal ligation and puncture (CLP) and in mouse C2C12 myoblasts through TNF-α treatment. *In vivo* experiments demonstrated decreased muscle strength, muscle fiber atrophy, widened intercellular spaces, and increased miR-542-3p/5p expression in ICU-AW mice model. *In vitro* experiments indicated suppressed ATG5, ATG7 and LC3II/I, elevated MDA and ROS levels, decreased SOD levels, and reduced MMP in the model group. Similar to animal experiments, the expression of miR-542-3p/5p was upregulated. Gel electrophoresis explored the binding of polyethyleneimine/mesoporous silica nanoparticles (PEI/MMNs) to locked nucleic acid (LNA) miR-542 inhibitor (LNA-542). PEI/MMNs@LNA-542 with positive charge (3.03 ± 0.363 mV) and narrow size (206.94 ± 6.19 nm) were characterized. Immunofluorescence indicated significant internalization with no apparent cytotoxicity. Biological activity, examined through intraperitoneal injection, showed that PEI/MMNs@LNA-542 alleviated muscle strength decline, restored fiber damage, and recovered mitochondrial injury in mice. In conclusion, PEI/MMNs nanoparticles effectively delivered LNA-542, targeting ATG5 to inhibit autophagy and alleviate mitochondrial damage, thereby improving ICU-AW.

## 1 Introduction

Intensive care unit-acquired weakness (ICU-AW) is a common complication in critically ill patients that involves changes in neurological and muscular function and structure. It is usually in the form of symmetrical muscle weakness, mainly in the respiratory and proximal limb muscles [1]. Clinically, majority of ICU-AW patients present with pulmonary complications, tetraplegia, shock, and difficulty in ventilating off the ventilator [2–4], resulting in reduced quality of life, extended hospital stay, and decreased survival rate [5]. Although early rehabilitation [6] and early activity [7] have been found to decrease the incidence of ICU-AW, activity limitation remains an issue. Therefore, there is a need for research aimed at developing innovative interventions for the management of ICU-AW.

Mounting evidence indicates that the expression of miRNAs is affected during the ICU-AW process, and miRNAs have the ability to regulate the progression of ICU-AW. MicroRNAs (miRNAs) are a new class of molecular targeting markers with a length of 19–25 nucleotides that regulate the expression of target genes by degrading target mRNAs or inhibiting protein translation [8]. Garros et al.’s research reveals an elevated expression level of miR-542 in ICU-AW patients [9]. In mouse experiments, miR-542 overexpression leads to muscle atrophy, diminished mitochondrial function, and increased mitochondrial ribosomal stress. Therefore, inhibiting the expression of miR-542 might be a potential strategy to alleviate the progression of ICU-AW. An important characteristic of the ICU-AW process is the degradation of muscle proteins, and several key pathways that play a crucial role in promoting muscle protein degradation including the ubiquitin-proteasome system (UPS), dysregulated autophagy, and mitochondrial dysfunction [10]. The autophagy-associated protein–protein interaction network has a crucial member, ATG5 protein, which is believed to play an essential role in the autophagic process [11,12]. Previous research has indicated that ATG5 is involved in the development and therapeutic mechanisms of numerous diseases, such as brain tumors [13], kidney injury [14], and liver injury [15]. The miR-542/ATG5 axis has been found to promote proliferation, migration, invasion, and autophagy of neuroblastoma cells [16]. Hence, further research is needed to elucidate the mechanistic role of miR-542 in ICU-AW.

MiRNA inhibitors have the capability to suppress local or systemic miRNA expression. However, due to their negative charge and short half-life, effectively delivering miRNA inhibitors to target cells remains a major challenge [17]. Nanoparticle delivery systems can effectively reduce the renal clearance rate of miRNA inhibitors, allowing nanoparticles to escape renal clearance, thereby significantly increasing drug concentration at the target site. This simultaneously minimizes adverse effects and promotes cellular uptake [18]. Nanoparticles for miRNA delivery include lipids and liposomes, polymer carriers, and inorganic nanoparticles [19]. Among them, cationic polymer polyethyleneimine (PEI) is one of the most widely used and studied polymers for gene delivery, given its positively charged surface that facilitates binding with negatively charged miRNA [20]. Mesoporous silica nanoparticles (MMN) constitute a nanocarrier system with a large pore surface area and excellent biocompatibility, efficiently loading active molecules [21]. Surface-loading PEI on MMNs enhances nucleic acid transfection efficiency and stability. To our knowledge, the potential of miR-542 inhibition in treating ICU-AW has not been explored. Therefore, in this study, we first investigate the changes and potential mechanisms of miR-542 in ICU-AW *in vivo* and *in vitro*. Subsequently, locked nucleic acid (LNA)-based miR-542 inhibitor (LNA-542) is encapsulated in MMNs and modified with PEI to synthesize nanoparticles, and the biological activity of these nanoparticles in alleviating ICU-AW is evaluated *in vivo*.

## 2 Materials and methods

### 2.1 Synthesis of MMNs

Weigh 1.0 g of hexadecyl trimethyl ammonium bromide and dissolve it in 160 mL of ultrapure water under stirring conditions at 35°C (200 rpm). After 15 min, add 3 mL of concentrated ammonia to form a homogeneous and transparent solution. While stirring, dropwise add 24 mL mixed solution (*n*-hexane:ethyl silicate = 5:1) into the reaction system, completing this step in approximately 30 min. Continue the reaction for 12 h at 35°C, during which the reaction system gradually transforms into a homogeneous milky-white colloidal solution. Collect the product by centrifugation for 10 min (4,000 rpm) and wash it several times with ultrapure water and anhydrous ethanol. Disperse the collected solid sample in 2.4 mL of 5 M HCl and 100 mL of anhydrous ethanol, reflux and stir at 90°C for 5 h, repeat the extraction process three times, and finally dry the sample in a vacuum oven to obtain MMNs.

### 2.2 Synthesis of PEI/MMNs@LNA-542

Weigh 200 mg of PEI and dissolve it in a 100 mL round-bottom flask using 10 mL of deionized water. Add 50 mg each of N-hydroxysuccinimide and 1-(3-dimethylaminopropyl)-3-ethylcarbodiimide hydrochloride and activate for 30 min. Disperse 200 mg of MMNs in 10 mL of deionized water, mix thoroughly with different amounts (200, 100, 50, 33, and 25 mg) of miR-542 inhibitor (LNA-542, RiboBio, China), and then add each mixture into the PEI solution for reaction. After incubating at room temperature for 4 h, perform overnight dialysis using a dialysis bag with a molecular weight cutoff of 5,000, collect the precipitate to obtain PEI/MMNs@LNA-542 at different concentrations. Determine the optimal concentration using gel electrophoresis. Synthesize PEI/MMNs@LNA-NC by replacing LNA-542 with LNA-NC using the same method.

### 2.3 Detection of binding capacity of PEI/MMNs nanoparticles with LNA-542

The binding capacity of PEI/MMNs nanoparticles with LNA-542 is investigated through gel electrophoresis. A 1% agarose gel (containing 0.2 μg/mL ethidium bromide) is used for electrophoresis. Inject 10 μL of different ratios of polymer/gene nanoparticles into the wells of the gel. Use 1× Tris–acetate–EDTA (TAE) buffer as the electrophoresis buffer, run the gel at 120 V for 25 min, and visualize the electrophoresis results using the BioDoc-ItTM System.

### 2.4 Morphology and potential characterization

Use transmission electron microscopy (TEM; FEI Talos F200X, USA) to characterize the morphology, microscopic structure, and composition of MMNs and PEI/MMNs@LNA-542. Use the Zetasizer Nano to measure the hydrated particle size and zeta potential of MMNs, PEI/MMNs, and PEI/MMNs@LNA-542.

### 2.5 Cell uptake study

We utilized a fluorescence-labeled miRNA inhibitor (FAM-LNA-542, RiboBio, China) for cellular uptake studies. Cellular uptake of PEI/MMNs@FAM-LNA-542 nanoparticles in C2C12 cells was assessed through fluorescence microscopy under conditions where the polymer-to-nucleic acid weight ratio was 6:1. C2C12 cells (6 × 10^4^ cells) were seeded in a 24-well plate and cultured overnight at 37°C to reach approximately 70% confluency at the time of delivery. Subsequently, the cells were co-cultured with the nanoparticles dispersion in Dulbecco phosphate buffer saline (100 µL) containing 1% serum in the culture medium at 37°C for cell uptake studies. The final concentration of FAM-LNA-542 in each well was 50 nM. After 6 h of incubation, cells were washed with PBS, trypsinized, and centrifuged at 1,500 rpm for 5 min. Visualization of the binding between PEI/MMNs nanoparticles and cells was performed using fluorescence microscopy (Zeiss, Axio Vert.A1). For comparison, naked FAM-LNA-542 (50 nM) was used as a negative control. Lipofectamine 2000 (Invitrogen) was employed as a positive control. The Lipofectamine 2000/FAM-LNA-542 complex was prepared according to the manufacturer’s protocol (Invitrogen).

### 2.6 ICU-AW animal model

A total of 12 C57BL/6 male mice (20–25 g) aged 10 weeks were purchased from Sipeifu Biotechnology Co., Ltd. The control group consisted of six animals that underwent a sham surgery, where a laparotomy was performed without any manipulation or alteration of the cecum. In contrast, the model group, also consisting of six animals, underwent a procedure to induce cecal ligation and perforation. The mice were randomly assigned for the experiments using a table of random numbers. Given the challenging nature of reconstructing an ICU-AW model, referencing previous research [22,23], the establishment methods for a sepsis model can be employed as a means to create an ICU-AW model. In short, anesthesia was administered to the mice in the model group via intraperitoneal injection of pentobarbital solution. Once the mice were successfully anesthetized, an incision was made on the skin to expose the cecum. The cecal mesentery was then dissected to approximately 50% of the cecum’s length and ligated using silk thread. A sterile empty needle was then used to penetrate both walls of the cecum, and its contents were expressed and wiped away. The appendix was then repositioned, and the abdomen was closed. Intraperitoneal saline and intramuscular pethidine were administered to the mice before awakening them and returning them to the surrogate room. The location of the cage was randomly assigned. The mice were injected with PEI/MMNs@LNA-NC and PEI/MMNs@LNA-542 intraperitoneally (at a dosage of 120 nmol/kg) every 2 days and were divided into two groups: the PEI/MMNs@LNA-NC group and PEI/MMNs@LNA-542 group. The animals were kept under a 12 h light–dark cycle and provided *ad libitum* access to food and water. For tissue collection, mice were euthanized and the diaphragm and gastrocnemius muscle were dissected, isolating them from the tendons. Half of the muscle was frozen and cut into 10 μm-thick frozen sections, while the other half was snap-frozen and stored at −80°C until further analysis. This study was approved by the Scientific Ethics Committee of Beijing Chaoyang Hospital, Capital Medical University (2022-d-216) on March 1, 2022. All outcome assessments were performed in a blinded manner.


**Ethical approval:** The research related to animal use has been complied with all the relevant national regulations and institutional policies for the care and use of animals, and has been approved by the Scientific Ethics Committee of Beijing Chaoyang Hospital, Capital Medical University (2022-d-216).

### 2.7 ICU-AW cell model

The establishment of the ICU-AW cell model has been refined based on previous research [24]. Mouse myoblasts cell C2C12 (m013, icell) were cultured in Dulbecco’s Modified Eagle Medium (DMEM) containing 20% newborn calf serum at 37°C with 5% CO_2_. The cells were then switched to DMEM supplemented with 2% heat-inactivated horse serum (Gibco) for 4 days to induce myoblast differentiation. Subsequently, the cells were treated with human recombinant tumor necrosis factor α (TNF-α; MCE, HY-P70426G). TNF-α was added to the differentiation medium every 24 h at a concentration of 5 ng/mL for a total of 4 days, establishing the model group. Cells not treated with TNF-α were designated as the control group.

### 2.8 Lennon score

Lennon’s score on changes in muscle strength of the mice at 1-week interval was as follows: 0 for normal muscle strength; 1 for insignificant rest and weakness in the limbs; 2 for mental inactivity and weakness in the forelimbs; 3 for muscle weakness, inability to grasp, or even difficulty in breathing; and 4 for near-death or death. A score of 0.5, 1.5, or 2.5 was assigned between the two manifestations.

### 2.9 Reactive oxygen species (ROS) detection assay

The samples from gastrocnemius and diaphragm were cut up and digested with 2 mg/mL collagenase type I. Next, the samples were placed in a 37°C oven for 30 min, followed by filtration with a 40 μm filter and centrifugation. The supernatant was removed and the remaining part of the precipitated cells was resuspended. Cells were collected and suspended in diluted DCFH-diacetate (DCFH-DA) and incubated for 20 min at 37°C in a cell incubator. The DCFH-DA probe and cells were mixed upside down to allow full contact, washed with serum-free cell culture medium to remove extracellular probes, and finally observed directly with a laser confocal microscope.

### 2.10 Hematoxylin/eosin (HE) staining

The frozen tissues of the gastrocnemius and diaphragm muscles were embedded, and sections were cut using a paraffin microtome. The sections were stained in Harris hematoxylin solution for 6 min, followed by removal of unbound hematoxylin. Subsequently, they were stained with eosin solution for approximately 1 min. After staining, the sections were rinsed with water until no further coloration occurred. Dehydration and permeabilization steps were performed, and the sections were finally sealed with neutral resin. The mean cross-sectional area (MCSA) of muscle fibers is quantified using ImageJ software, as previously described [25,26].

### 2.11 Superoxide dismutase (SOD) activity

The SOD activities of gastrocnemius and diaphragm were detected by SOD Activity Assay Kit (Solarbio life science). Briefly, 0.1 g of gastrocnemius and diaphragm tissues were weighed and combined with 1 mL of extraction solution. The mixture was then centrifuged at 8,000*g* for 10 min at 4°C, resulting in the collection of the supernatant. The solution in the reagent kit was thoroughly mixed, and then incubated in a 37°C water bath for 30 min. Finally, the absorbance value was measured at 560 nm.

### 2.12 Malondialdehyde (MDA) level

The MDA level of gastrocnemius and diaphragm were detected by MDA Content Assay Kit (Solarbio life science). Briefly, 0.1 g of tissues from gastrocnemius and diaphragm were weighed and combined with 1 mL extraction solution for ice bath homogenization, respectively. It was then centrifuged at 8,000*g* for 10 min at 4°C, the supernatant was removed, and the enzyme marker (Thermo Labsystems) was preheated for 40 min. The mixture of MDA assay working solution, sample, and reagent III (provided in the MDA assay kit) was kept in a water bath at 100°C for 30 min and then cooled in an ice bath and centrifuged for 10 min. The 200 µL of supernatant was pipetted into a 96-well plate and the absorbance of each sample was measured at 450, 532, and 600 nm.

### 2.13 RT-qPCR

RNA was extracted from cells using Trizo (Invitrogen) following the manufacturer’s protocol. The RNA concentration was determined using a spectrophotometer (Shanghai Sun Yu Heng Scientific Instrument Co., Ltd). In an ice bath, total RNA, 5× TransScript All-in-One SuperMix for qRT-qPCR, gDNA Remover, and RNase-free ddH_2_O were added to nucleic acid-free RT-qPCR tubes, mixed, and incubated at 42°C for 15 min. After diluting the cDNA samples ten times, they were used as detection templates. The 96-well plate containing the samples was placed in the ABI StepOne Plus fluorescence RT-qPCR instrument for the reaction. U6 were applied as an internal standard. PCR amplification was quantitated using the 2^−ΔΔCt^ method.

### 2.14 Mitochondrial membrane potential (MMP) measurements (JC-1 staining)

The amount of JC-1 staining working solution required for each well of a six-well plate was 1 mL. For cell suspensions, 0.5 mL JC-1 staining working solution was required per 500-1 million cells. Ultrapure water was added to dilute JC-1 and vortexed to fully dissolve and mix JC-1. Then JC-1 staining buffer (5×) was added and mixed well to make the JC-1 staining working solution. The carbonyl cyanide M-chlorophenylhydrazone (CCCP, 10 mM) provided in the kit (Beyotime) was set as a positive control. After that, 0.5% trypsin was digested and resuspended in 0.5 mL of cell culture medium, and 0.5 mL of JC-1 staining working solution was added and mixed well. The cells were incubated for 20 min at 37°C in the cell incubator and then centrifuged at 600*g* for 3–4 min at 4°C. Next, they were precipitated, and the supernatant was discarded, washed twice with JC-1 staining buffer (1×), and resuspended with an appropriate amount of JC-1 staining buffer (1×) before analysis by flow cytometry (Beckman Coulter).

### 2.15 Western blot assay

Cell lysis was initiated using Lysis buffer (Beyotime) following the manufacturer’s instructions. The extracted protein supernatant was boiled and denatured, and protein separation was performed based on molecular size using polyacrylamide gel electrophoresis. Subsequently, proteins were transferred from the gel to a PVDF membrane (IPVH00010, Merck-Millipore). After membrane blocking, it was incubated overnight at 4°C with specific primary antibodies, including ATG5 (1:2,000; 10181-2-AP, Proteintech), ATG7 (1:2,000; 10088-2-AP, Proteintech), LC3 (1:2,000; 81004-1-RR, Proteintech), MuRF1 (1:5,000; 55456-1-AP, Proteintech), MAfbx (1:15,000; 67172-1-Ig, Proteintech), and GAPDH (1:10,000; 10494-1-AP, Proteintech). Subsequently, it was incubated at 37°C for 2 h with HRP-goat anti-rabbit recombinant secondary antibody (1:10,000; RGAR001, Proteintech). Detection was performed using ECL, and blot quantification was carried out using ImageJ software (NIH, Bethesda, MD, USA).

### 2.16 Statistical analysis

The statistical analysis was performed using PRISM (version 5.01, GraphPad). The data were expressed as mean ± standard deviation. The significance of the differences between two groups was determined using the Student’s *t*-test. For the comparison of multiple groups, one-way analysis of variance was utilized. A *p*-value of less than 0.05 was considered to indicate statistical significance.

## 3 Results

### 3.1 Mechanism of miR-542/ATG5 axis in ICU-AW mice

The muscle strength and gastrocnemius/body weight of the hind limbs of the mice were examined every 7 days in the normal group (*n* = 6) and the model group (*n* = 6). As shown in Table 1, the muscle strength scores of the model group increased significantly on Days 7 and 14 compared to the control group (*P* < 0.01), and the longer the time, the higher the muscle strength scores. In addition, the gastrocnemius/body weight values of the model group were significantly lower on Days 7 and 14 compared to the control group (*P* < 0.01), and the longer the time, the lower the gastrocnemius/body weight values (Table 2). Thus, it is evident that the mice in the model group exhibited muscle weakness and atrophy.

**Table 1:** Muscle strength of control group and model group

Group	*n*	Muscle strength scores		
		0 day	7 days	14 days
Normal	6	0.000 ± 0.000	0.000 ± 0.000	0.000 ± 0.000
Model	6	0.000 ± 0.000	1.667 ± 0.816**	3.000 ± 0.632**

^**^The muscle strength scores of the model group increased significantly on days 7 and 14 compared to the control group (*P* < 0.01).

**Table 2:** Gastrocnemius and body weight of control group and model group

Group	*n*	Gastrocnemius/body weight		
		0 day	7 days	14 days
Control	6	0.0156 ± 0.0002	0.0157 ± 0.0002	0.0157 ± 0.0005
Model	6	0.0152 ± 0.0001	0.0124 ± 0.0003**	0.0098 ± 0.0005**

^**^The gastrocnemius/body weight values of the model group were significantly lower on days 7 and 14 compared to the control group (*P* < 0.01).

**Figure 1:**
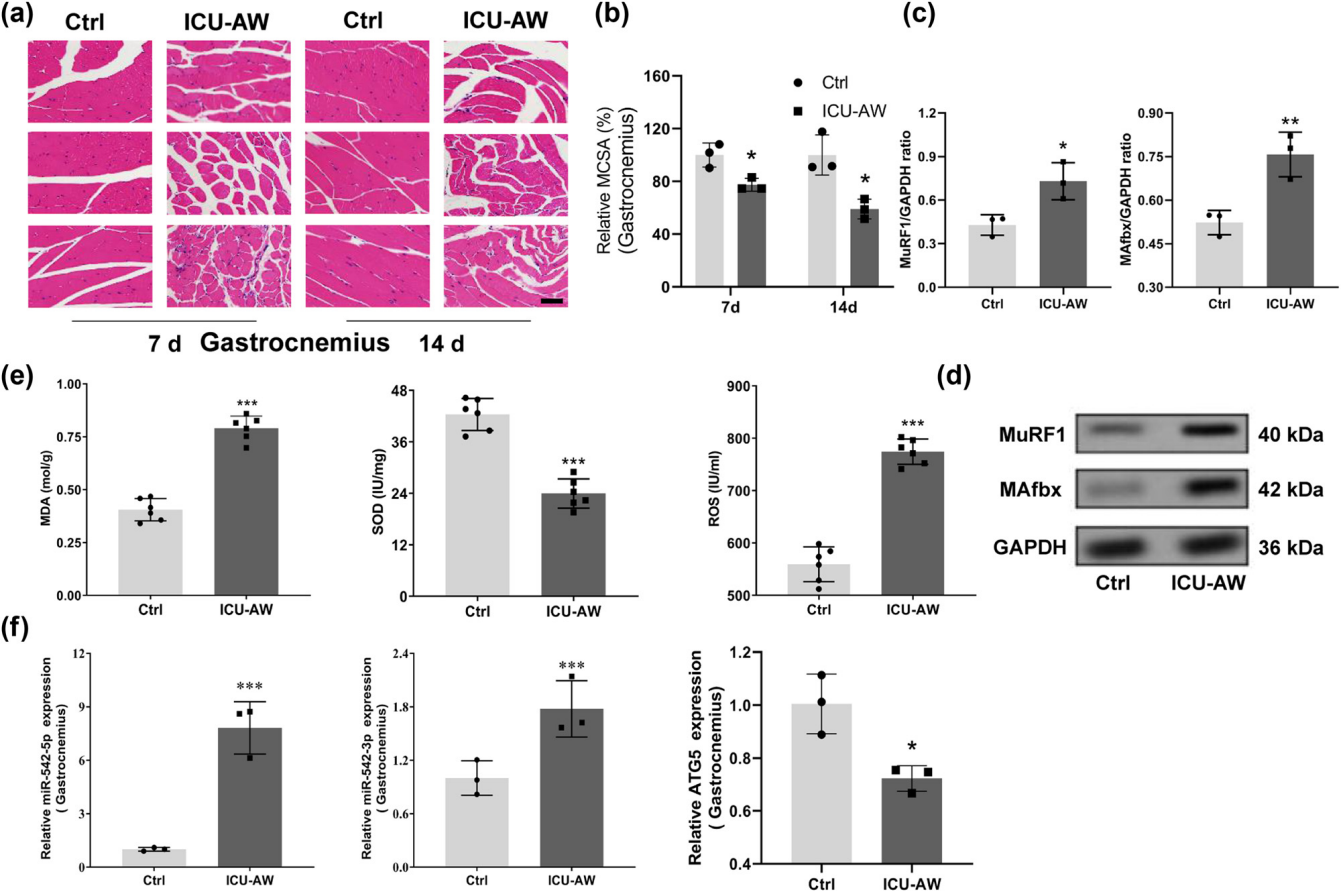
Changes of the gastrocnemius in ICU-AW mice. (a) The pathological changes of the gastrocnemius in control and ICU-AW animal groups at 7 and 14 days were observed by HE staining. (b) MCSA of gastrocnemius muscle. (c) and (d) WB assay was applied to detect MAFbx and MuRF1 expression. (e) The MDA levels, mitochondrial ROS levels, and SOD activities of the gastrocnemius in both groups were examined. (f) RT-qPCR was performed to detect the levels of miR-542 and ATG5 in the gastrocnemius muscle of the control and ICU-AW animal groups. *P < 0.05, ***P < 0.001, compared with control group.

**Figure 2:**
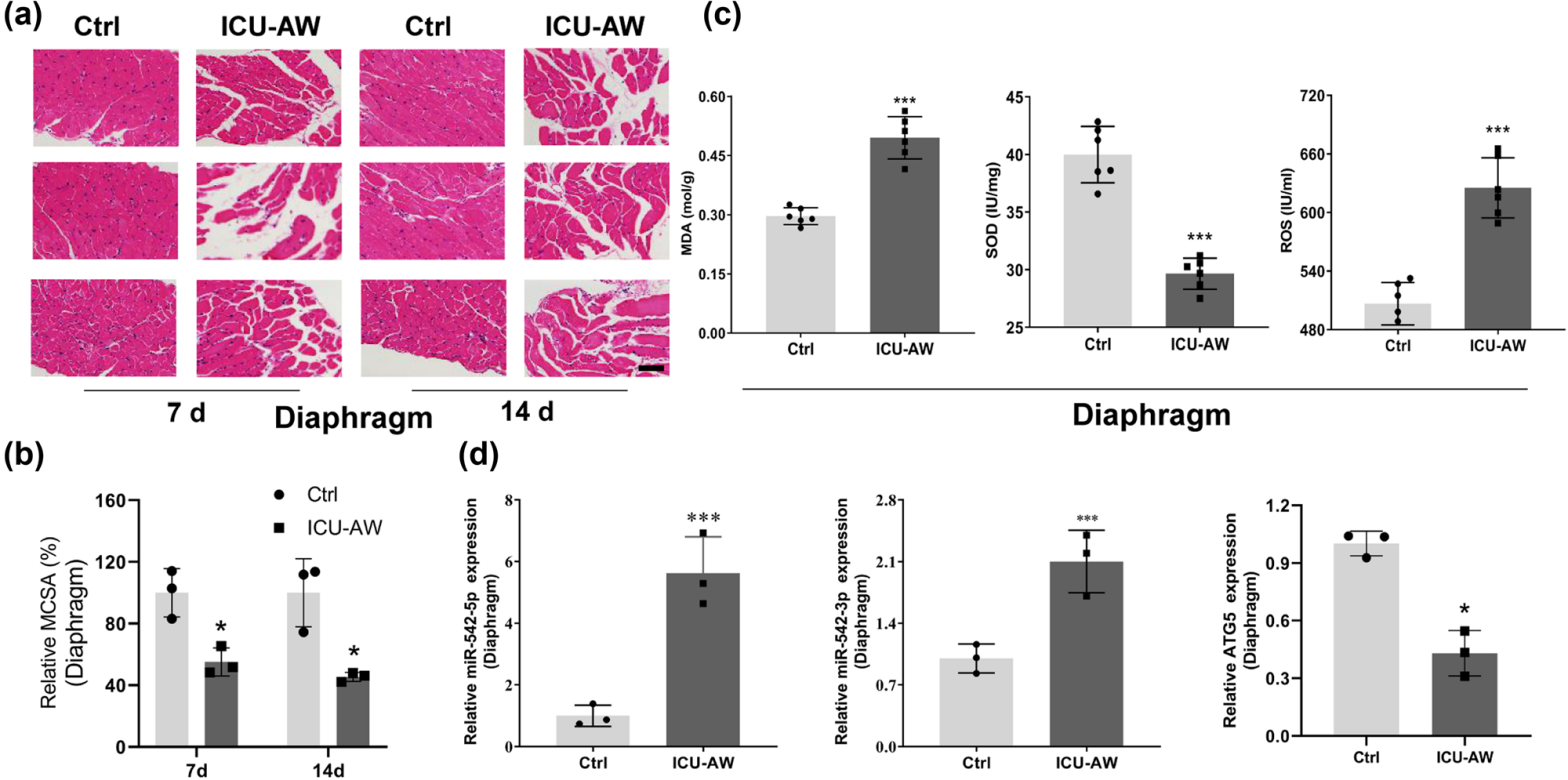
Changes of the diaphragm muscle in ICU-AW mice. (a) The pathological changes of the diaphragm muscle in control and ICU-AW animal groups at 7 and 14 days were observed by HE staining. (b) MCSA of diaphragm muscle. (c) The MDA levels, mitochondrial ROS levels, and SOD activities of the diaphragm in both groups were examined. (d) RT-qPCR was performed to detect the levels of miR-542 and ATG5 in the diaphragm muscle of the control and ICU-AW animal groups. *P < 0.05, ***P < 0.001, compared with control group.

Pathological changes in the gastrocnemius and diaphragm at 7 and 14 days were examined by HE staining, as shown in Figures 1a and 2a. The nuclei were purple-blue and the cytoplasm and extracellular matrix were red. The myocytes in normal tissues had a regular polygonal morphology and are neatly arranged and uniform in size, with multiple nuclei located under the muscle membrane. In the model group, muscle cells exhibit partial muscle fiber atrophy, widened interstitial spaces between myofibers, multiple regions with lighter staining of sarcoplasm, partial disappearance of striations, and the pathological changes in the gastrocnemius and diaphragm muscles become more severe over time. The MCSA of the gastrocnemius and diaphragm in the model group was significantly lower than in the control group (*P* < 0.05, Figures 1b and 2b). The UPS, a protein degradation pathway, plays a crucial role in skeletal muscle atrophy, with increased expression of the E3 ubiquitin ligases MAFbx and MuRF1 genes in atrophied skeletal muscles [27]. Western blot results indicated a significant increase in the expression of MuRF1 and MAFbx in the gastrocnemius of the ICU-AW group, suggesting atrophy in the gastrocnemius of ICU-AW group mice (Figure 1c and d). In addition, MDA levels and SOD levels were significantly higher (*P* < 0.001) while mitochondrial ROS levels were significantly lower (*P* < 0.001) in the model group compared to the control group (Figures 1e and 2c).

The expression levels of miR-542-3p and miR-542-5p in the diaphragm and gastrocnemius muscle were examined by RT-qPCR and were found to be prominently elevated in the model group compared to the control group (*P* < 0.001) (Figures 1f and 2d). Additionally, ATG5 expression levels of the diaphragm and gastrocnemius were significantly lower in the model groups compared to the control group (*P* < 0.05) (Figures 1f and 2d).

### 3.2 Mechanism of miR-542/ATG5 axis in ICU-AW cells

**Figure 3:**
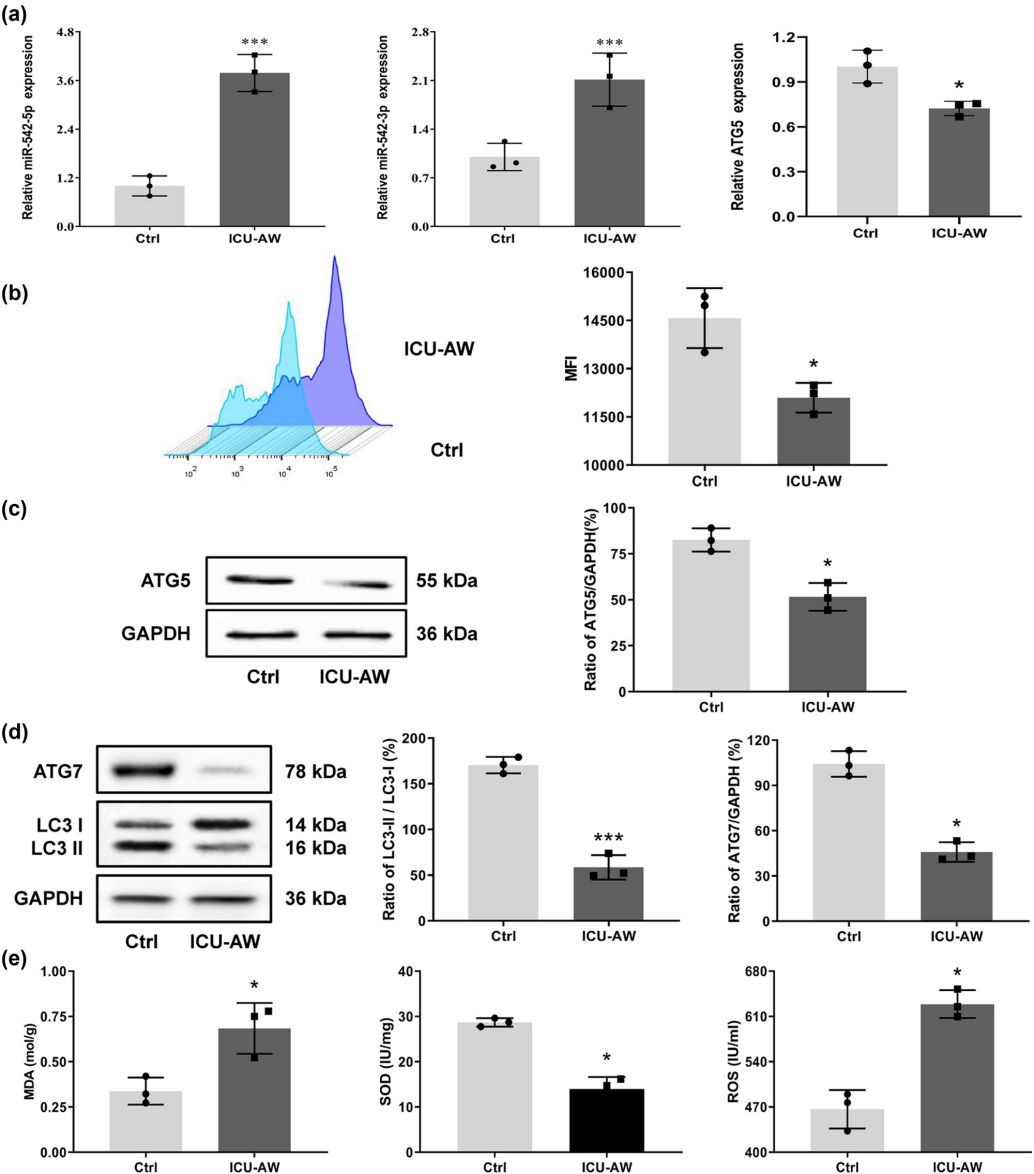
Changes of miR-542, ATG5, and MMP in ICU-AW cell model. (a) RT-qPCR was performed to detect the levels of miR-542 and ATG5 in the control and ICU-AW cell groups. (b) MMP changes were detected using the JC-1 fluorescent probe method. (c) and (d) WB assay was applied to detect autophagy-associated protein expression and (e) protein expression related to mitochondrial damage in the control and ICU-AW cell groups. *P < 0.05, ***P < 0.001, compared with control group.

The levels of miR-542 and ATG5 in the two groups of cells were examined by RT-qPCR, and it was found that the expression levels of miR-542-3p and miR-542-5p were dramatically increased (*P* < 0.001) in the model group, while the expression levels of ATG5 were significantly decreased (*P* < 0.01) compared to the control group (Figure 3a). This was consistent with the results of the animal model. In addition, mitochondria were extracted from the tissues of each group, and the changes in MMP were detected using the JC-1 fluorescent probe method. Results showed that the mean fluorescence intensity was significantly lower (*P* < 0.05) in the model group compared with the control group (Figure 3b). It can be concluded that the mitochondrial function of ICU-AW cells was diminished.

The expressions of autophagy-related proteins and mitochondrial autophagy-related proteins were examined via WB. The expressions of LC3II/LC3I, ATG5, and ATG7 were significantly lower in the model group compared to the control group (*P* < 0.05) (Figure 3c and d). In contrast, MDA levels and mitochondrial ROS levels were prominently raised (*P* < 0.05), and SOD activity was significantly lessened (*P* < 0.05) (Figure 3e). Hence, it is evident that the autophagy level in ICU-AW cells is reduced while mitochondrial damage is increased.

### 3.3 Characterization of PEI/MMNs@LNA-542

**Figure 4:**
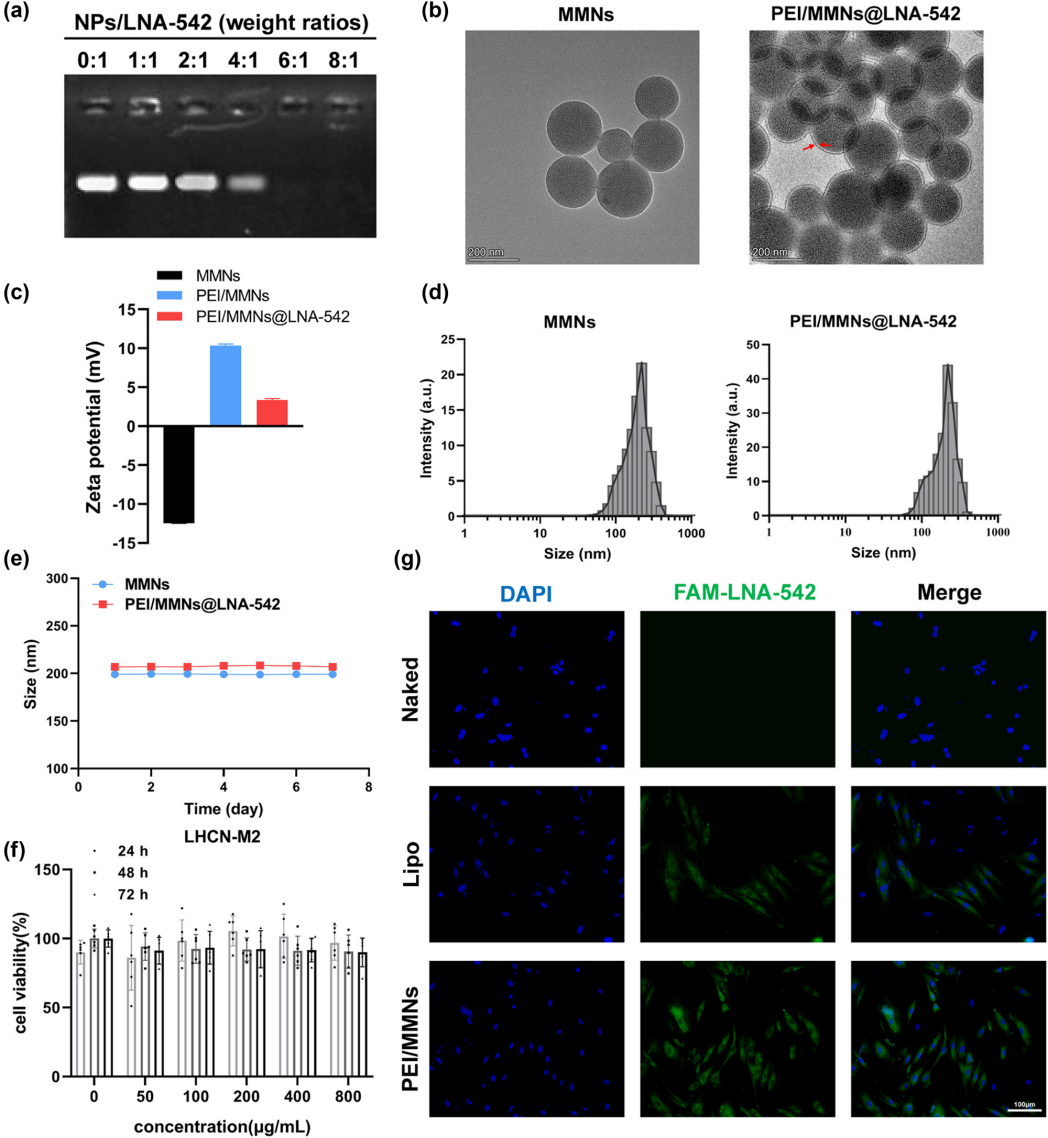
Characterization of PEI/MMNs@LNA-542. (a) Gel electrophoresis experiment to assess the binding capacity of nanoparticles and LNA-542. (b) TEM images. (c) ζ potential. (d) Size distributions of MMNs and PEI/MMNs@LNA-54 NPs. (e) Changes in particle size within 7 days after the construction of PEI/MMNs@LNA-54 NPs. (f) Impact of nanoparticles at different concentrations on the viability of C2C12 cells at different time points. (g) Immunofluorescence assay to evaluate the uptake ability of C2C12 cells for PEI/MMNs@FAM-LNA-542, with naked FAM-LNA-542 as the negative control and Lipofectamine 2000/FAM-LNA-542 complex as the positive control.

PEI/MMNs@LNA-542 was successfully synthesized. Electrophoretic mobility measurements were used to detect the formation of polyelectrolyte nanoparticles between PEI/MMNs and LNA-542. When the weight ratio of NPs to LNA-542 was 6:1, LNA-542 did not migrate, indicating complete binding between PEI/MMNs and LNA-542 (Figure 4a). Subsequently, a weight ratio of 6:1 for NPs to LNA-542 was adopted for the synthesis of PEI/MMNs@LNA-542. TEM results show that MMNs nanoparticles are spherical with mesopores, and after PEI modification, the nanoparticles maintain a relatively regular circular shape with visible PEI coating on the surface (Figure 4b). The average particle size of MMNs nanoparticles was measured to be 199.00 ± 0.62 nm, and for PEI/MMNs@LNA-542, it was 206.94 ± 6.19 nm (Figure 4c). The average zeta potential of MMN nanoparticles was −12.46 ± 0.04 mV, indicating a negative charge. After PEI modification, the nanoparticles became positively charged, and the potential slightly decreased after loading negatively charged LNA-542, confirming successful encapsulation (Figure 4d). Continuous measurement of the average particle size of nanoparticles in water for 7 days showed no significant changes, indicating good stability (Figure 4e).

The cytotoxicity of empty nanoparticles PEI/MMNs on C2C12 cells was assessed. After exposure to different concentrations for varying times, cell viability remained above 80%, suggesting that cell growth and quantity were not significantly affected. This indicates that the prepared nanoparticles exhibit excellent biocompatibility (Figure 4f). The uptake of PEI/MMNs@LNA-542 nanoparticles by C2C12 cells was investigated using FAM-labeled LNA-542 (FAM-LNA-542). Naked FAM-LNA-542 (50 nM) served as a negative control, and the commonly used transfection lipid reagent Lipofectamine 2000 served as a positive control. Results indicate that PEI/MMNs@LNA-542 nanoparticles are more readily taken up by C2C12 cells.

### 3.4 PEI/MMNs@LNA-542 alleviates ICU-AW mouse injuries by restoring mitochondrial damage

In order to further explore the *in vivo* efficacy of PEI/MMNs@LNA-542 nanoparticles in restoring ICU-AW, PEI/MMNs@LNA-542 nanoparticles were administered via intraperitoneal injection into C57BL/6 mice. As shown in Table 3, the muscle strength scores of the model and PEI/MMNs@LNA-NC groups at 7 and 14 days were significantly higher (*P* < 0.01) compared to the control group, while the muscle strength scores of the PEI/MMNs@LNA-542 group were remarkably lower (*P* < 0.01) compared to the PEI/MMNs@LNA-NC group. A higher muscle strength score indicates weaker muscle strength. As shown in Table 4, the gastrocnemius/body weight values at 7 and 14 days (*P* < 0.01) were prominently reduced in the model and PEI/MMNs@LNA-NC groups compared to the control group, while the gastrocnemius/body weight values were notably elevated in the PEI/MMNs@LNA-542 group compared to the PEI/MMNs@LNA-NC group (*P* < 0.01).

**Table 3:** Muscle strength of PEI/MMNs@LNA-542 group and PEI/MMNs@LNA-NC group

Group	*n*	Muscle strength scores		
		0 day	7 days	14 days
Ctrl	6	0.000 ± 0.000	0.000 ± 0.000	0.000 ± 0.000
Model	6	0.000 ± 0.000	1.883 ± 0.752**	2.833 ± 0.752**
PEI/MMNs@LNA-NC	6	0.000 ± 0.000	2.167 ± 0.408**	3.167 ± 0.752**
PEI/MMNs@LNA-542	6	0.000 ± 0.000	1.333 ± 0.516^^##^^	1.833 ± 0.752^^##^^

A score of 0 indicates normal muscle strength; a score of 1 suggests no apparent rest weakness but limb weakness; a score of 2 indicates lethargy and front limb weakness; a score of 3 reflects muscle weakness, the inability to grasp or breathe difficulties; and a score of 4 denotes impending death or death. Scores between these manifestations are assigned values of 0.5, 1.5, or 2.5. ^**^The muscle strength scores of the model and PEI/MMNs@LNA-NC groups were significantly higher (*P* < 0.01) at 7 and 14 days compared to the control group. ^##^The muscle strength scores of the PEI/MMNs@LNA-542 group were significantly lower (*P* < 0.01) compared to the PEI/MMNs@LNA-NC group.

**Table 4:** Gastrocnemius/body weight of PEI/MMNs@LNA-542 and PEI/MMNs@LNA-NC group

Group	*n*	Gastrocnemius/body weight		
		0 day	7 days	14 days
Normal	6	0.0157 ± 0.0002	0.0159 ± 0.0003	0.0160 ± 0.0002
Model	6	0.0152 ± 0.0004	0.0123 ± 0.0004**	0.0097 ± 0.0005**
PEI/MMNs@LNA-NC	6	0.0158 ± 0.0002	0.0123 ± 0.0005**	0.0097 ± 0.0008**
PEI/MMNs@LNA-542	6	0.0152 ± 0.0003	0.0145 ± 0.0006^##^	0.0134 ± 0.0005^^##^^

^**^The gastrocnemius/body weight values were significantly lower in the model and PEI/MMNs@LNA-NC group compared to the control group at 7 and 14 days (*P* < 0.01). ^##^The gastrocnemius/body weight values were significantly higher in the PEI/MMNs@LNA-542 group compared to the PEI/MMNs@LNA-NC group (*P* < 0.01).

**Figure 5:**
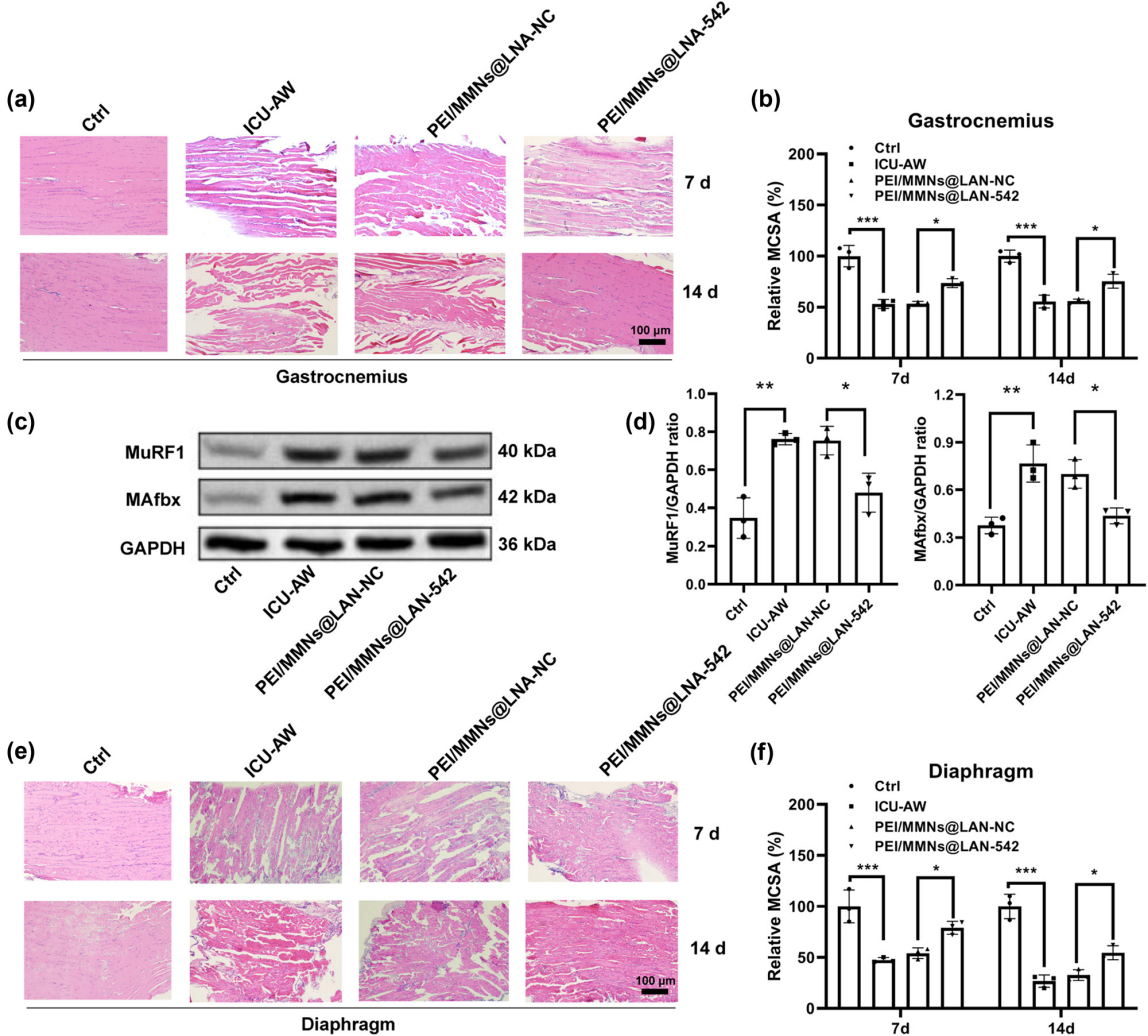
Contribution of PEI/MMNs@LNA-542 NPs to the gastrocnemius and diaphragm of ICU-AW mice. (a) HE staining was performed to observe the pathological changes in the gastrocnemius muscles at 7 and 14 days. (b) MCSA of gastrocnemius muscle. (c) WB assay was applied to detect MAFbx and MuRF1 expression. (d) Quantification of WB analysis for MAFbx and MuRF1. (e) HE staining was performed to observe the pathological changes in the diaphragm muscle at 7 and 14 days. (f) MCSA of diaphragm muscle. *P < 0.05, **P < 0.01, ***P < 0.001.

As shown in Figure 5a and e, compared to the control group, pathological changes in the gastrocnemius and diaphragm muscles were observed in the model group and PEI/MMNs@LNA-NC group at 7 and 14 days through HE staining. These changes included partial muscle fiber atrophy, widened intercellular spaces of striated muscle cells, regions of lighter muscle staining, and partial loss of striations. By quantifying the CSA of muscle cells, it is evident that the model group experienced muscle cell atrophy, whereas the PEI/MMNs@LNA-542 group showed an increase in muscle cell MCSA (Figure 5b and f). Furthermore, PEI/MMNs@LNA-542 treatment inhibited the CLP-induced increase of MuRF1 and MAFbx in muscle tissue (Figure 5c and d).

**Figure 6:**
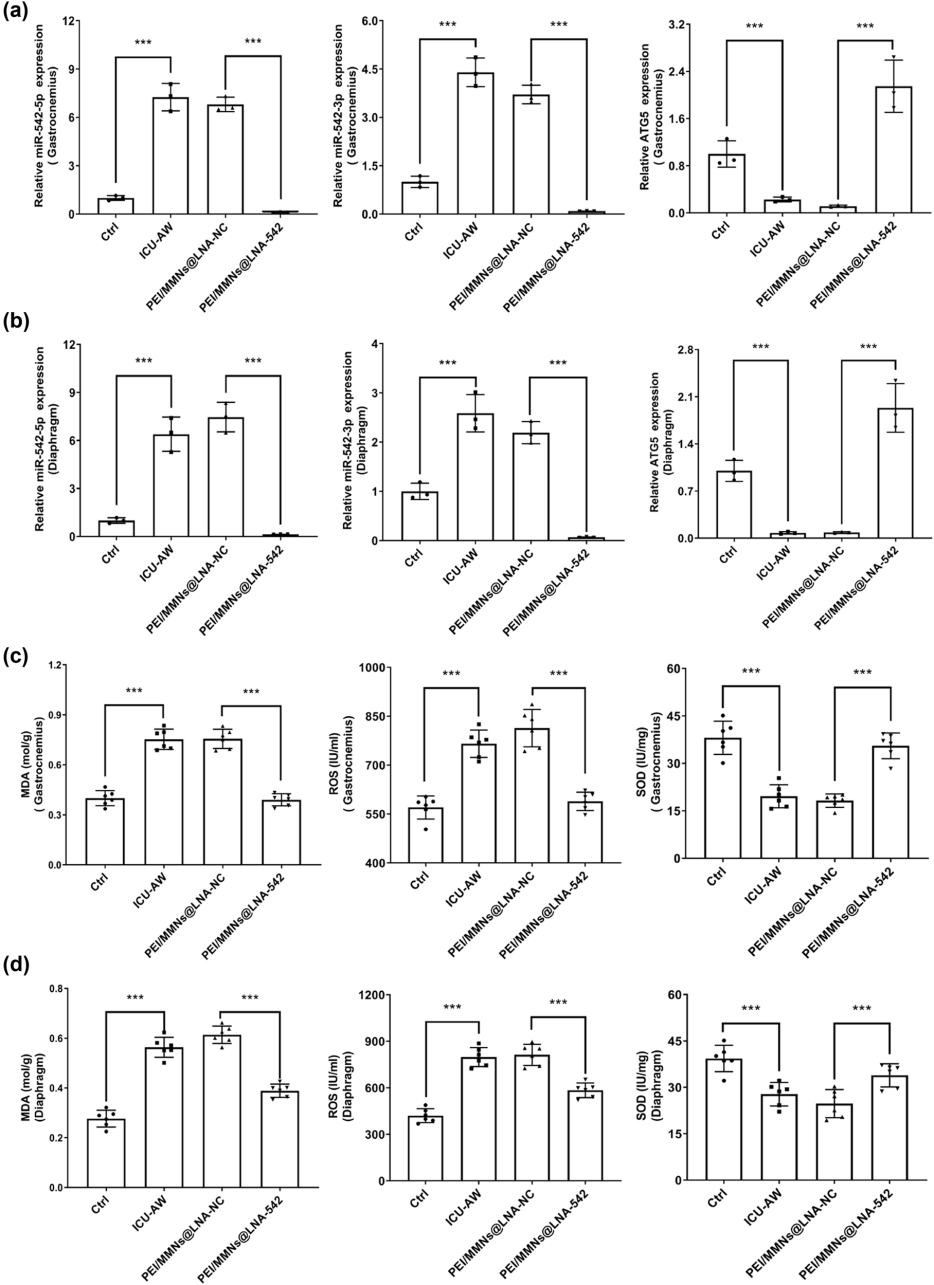
PEI/MMNs@LNA-542 NPs regulates miR-542, ATG5, and mitochondrial damage in ICU-AW mice. RT-qPCR assay of miR-542 and ATG5 levels in the gastrocnemius (a) and diaphragm (b) of the control group, ICU-AW group, PEI/MMNs@LNA-NC group, and PEI/MMNs@LNA-542 group. MDA levels, mitochondrial ROS levels, and SOD activity were measured in the gastrocnemius (c) and diaphragm (d) muscles of the control group, ICU-AW group, PEI/MMNs@LNA-NC group, and PEI/MMNs@LNA-542 group. ***P < 0.001.

In addition, the changes of miR-542 in the gastrocnemius and diaphragm of ICU-AW mice were examined by RT-qPCR. Results showed that the miR-542 expression levels were significantly increased in the gastrocnemius and diaphragm compared to the model group (*P* < 0.001), while the miR-542 expression levels were markedly lower in the PEI/MMNs@LNA-542 group compared to the PEI/MMNs@LNA-NC group (*P* < 0.001) (Figure 6a and b). Furthermore, the miR-542/ATG5 expression levels were dramatically reduced in the gastrocnemius and diaphragm model groups compared to the control group (*P* < 0.001), while the miR-542 expression levels were notably raised in the PEI/MMNs@LNA-542 group compared to the PEI/MMNs@LNA-NC group (*P* < 0.001) (Figure 6a and b). Thus, it is evident that miR-542 inhibited the expression of ATG5.

In the gastrocnemius and diaphragm, MDA levels and mitochondrial ROS levels were significantly increased (*P* < 0.001), and SOD activity was prominently lessened (*P* < 0.001) in the model group compared to the control group. And the two levels were remarkably lowered (*P* < 0.001) in the PEI/MMNs@LNA-542 group compared to the PEI/MMNs@LNA-NC group, while SOD activity dramatically increased (*P* < 0.001) (Figure 6c and d).

## 4 Discussion

ICU-AW is a prevalent acute neuromuscular dysfunction observed in critically ill patients, characterized by the absence of clear etiology. Elevated expression of miR-542 is identified in ICU-AW patients, and the heightened expression of miR-542 is implicated in the onset of ICU-AW, suggesting a potentially pivotal role for miR-542 in ICU-AW pathology. Our research findings indicate that the miR-542/ATG5 axis is activated during the pathological progression of ICU-AW, subsequently inhibiting cellular autophagy and exacerbating mitochondrial damage. To explore the therapeutic effects of miR-542 inhibition, PEI/MMNs@LNA-542 nanoparticles were successfully synthesized for miR-542 inhibition treatment. The PEI-coated nanoparticles exhibited regular circular morphology. The average particle size of PEI/MMNs@LNA-542 was measured at 206.94 ± 6.19 nm, with an average zeta potential of 3.03 ± 0.363 mV, demonstrating favorable stability and cellular uptake capabilities. *In vivo* experiments confirmed the capacity of PEI/MMNs@LNA-542 to mitigate the progression of ICU-AW.

Sepsis significantly impairs muscle function and protein synthesis in critically ill ICU patients, concurrently representing a major risk factor for ICU-AW. Due to the challenging reconstruction of an ICU-AW model, we established a CLP mouse model to simulate ICU-AW, as referenced from previous research [25]. Although it is not the most ideal model, the results demonstrate its confidentiality to simulate muscle tissue atrophy and weakness in ICU-AW model animals. Using a more reliable ICU-AW animal model might produce results that better reflect real-world conditions. Considering the substantial impact of inflammatory mediators, including TNF-α, during the ICU-AW process, TNF-α regulates NF-κB, promoting muscle atrophy and skeletal muscle protein loss [28]. This mechanism results in changes similar to those observed in the skeletal and respiratory muscles during ICU-AW. Therefore, in this study, we constructed an ICU-AW cell model by incubating TNF-α with C2C12 cells for 4 days. In this study, the cell model primarily investigated autophagy, MMP, and oxidative stress in ICU-AW using C2C12 cells. The results indicated that under ICU-AW conditions, autophagy in C2C12 cells was suppressed, while mitochondrial damage and oxidative stress were increased.

Muscular atrophy, characterized by the decline of skeletal muscle mass and function, is a crucial factor that contributes to ICU-AW [29]. The muscular strength of the ICU-AW animal model notably diminishes, particularly in limb muscles, as evidenced by muscle strength assessments (Table 1). This study also included the detection of UPS-related proteins. The WB results indicate an increased expression of MAFbx and MuRF1 in the gastrocnemius muscle of ICU-AW mice, suggesting skeletal muscle atrophy in these mice. The muscle mass-to-body weight ratios (Tables 2 and 4) further reflect atrophy of the gastrocnemius and diaphragm muscle. Jung et al. indicated that the main consequence of ICU-AW is diaphragmatic weakness [30] and they found diaphragmatic dysfunction in most ICU-AW patients using a multimodal approach. The current trial examined the gastrocnemius and diaphragm muscles and found severe myocyte lesions, with a significant reduction in myocyte area, and significantly increased miR-542 expression levels in the model group, potentially attributable to the inhibition of ribosome and protein synthesis by miR-542 [31]. It has been demonstrated that inhibition of the muscle miRNAs expression promotes muscle atrophy in ICU-AW [32]. Additionally, miR-542 is associated with neurodegenerative disease disorders [33]. This experiment discerns that inhibiting miR-542 can restore muscle strength and muscle tissue in the gastrocnemius and diaphragm of the ICU-AW animal model.

To investigate the underlying mechanisms, we constructed an ICU-AW cell model. ATG5, an essential protein for autophagy, plays an extensive role in detecting autophagy in myocytes [34–36]. Wen et al. illuminated that spongy miR-542 mediated the expression of ATG5 and promoted autophagy in tumor cells [16]. Furthermore, the present study, along with similar findings found in the study of Luo et al. [37], came to the conclusion that the miR-542/ATG5 axis could inhibit cellular autophagy. ATG7 is also a critically important autophagy-related protein. ATG7 knockout mice exhibit more severe mitochondrial dysfunction and muscle atrophy during sepsis [38]. In our cellular experiments, the expression of autophagy-related proteins ATG7 and LC3II/LC3I was significantly reduced in the ICU-AW group. Mitochondria play an important role in regulating energy metabolism in the control of physiological and pathological processes such as cellular autophagy and are critical for the therapeutic control of degenerative diseases [39]. *In vivo* experiments revealed that miR-542 induces mitochondrial damage and promotes ICU-AW. Likewise, the research by Garros et al. indicated that elevated miR-542-3p/5p may contribute to ICU-AW by promoting mitochondrial dysfunction [9]. A previous study suggested that autophagy inhibition leads to massive ROS release following impaired mitochondrial accumulation [40]. At the same time, excessive ROS release can regulate the process of mitochondrial autophagy [41] and can also impair muscle contractile function [42]. MDA is an important marker of oxidative stress [43], while oxidative stress and mitochondrial dysfunction appear to play a key role in muscle damage [44]. In *in vitro* experiments, autophagy was found to be inhibited and led to mitochondrial damage in the model group, while MDA levels and mitochondrial ROS levels were notably increased. Conversely, in *in vivo* experiments, inhibiting miR-542 promotes autophagy, alleviates mitochondrial damage, and exerts a rehabilitative effect on ICU-AW.

Research indicates that miRNA-181a can serve as a biomarker for diagnosing and predicting ICU-AW [45], promoting mitochondrial dysfunction and inflammatory response in ICU-AW rat models by inhibiting IGFBP5 expression [46]. This study discovered at both animal and cellular levels that miR-542 targets ATG5, promoting autophagy and exacerbating mitochondrial damage. This suggests that miRNA holds significant potential not only in the diagnosis and prediction of ICU-AW but also in its mitigation or treatment. Chemically modified miRNA inhibitors offer various advantages, including enhanced cellular uptake and transfection efficacy [47]. However, most chemically modified miRNA inhibitors exhibit a short *in vivo* half-life [48], necessitating high doses of oligonucleotides for *in vivo* inhibition, thereby increasing the risk of off-target effects. ‘Naked’ miRNA injection without assistance typically yields suboptimal results, but the use of cationic polymers may enhance this process [49,50]. To enhance the transfection efficiency of miRNA therapy, an efficient polymer carrier is required to bind and compress negatively charged RNA molecules into positively charged nanoparticles [51], increasing stability against enzymatic degradation, promoting cellular uptake, and allowing for substantial reduction in therapeutic dosage. For instance, research has shown that delivering exogenous miR-194 through gelatin nanospheres can effectively alleviate muscle atrophy [52]. In this study, we utilized PEI/MMNs conjugates for the *in vivo* and *in vitro* delivery of LNA-based miR-542 inhibitors, investigating the potential of PEI/MMN@LNA-542 nanoparticles to alleviate ICU-AW. PEI/MMN@LNA-542 nanoparticles exhibit a positive net charge (3.03 ± 0.363 mV) and a narrow size distribution (206.94 ± 6.19 nm), facilitating better internalization and absorption. Moreover, according to cytotoxicity assays, PEI/MMNs nanoparticles did not induce significant cytotoxicity, further illustrating their practicality and safety as carriers for miRNA inhibitors. Cellular uptake is a key parameter influencing the efficacy of non-viral gene carriers [53]. In cellular uptake experiments, we observed a significant increase in intracellular LNA-542 with PEI/MMNs nanoparticles compared to Lipofectamine. This may be attributed to the formation of more compact, stable, and easily binding extracellular nanoparticles by PEI/MMNs with miRNA inhibitors, enhancing interactions with the cell membrane and facilitating more efficient cellular uptake compared to Lipofectamine.

To assess the *in vivo* ability of PEI/MMN@LNA-542 nanoparticles to alleviate ICU-AW, we administered them via intraperitoneal injection. *In vivo* studies indicate that PEI/MMNs@LNA-542 can restore muscle tissue degradation and normal structure and can recover mouse muscle strength. PEI-modified MMNs nanoparticles binding miRNA inhibitors represent a drug delivery system for RNAi therapy, mitigating the progression of ICU-AW through the delivery of miR-542 inhibitors. However, introducing this delivery system for miR-542 inhibition therapy into clinical practice still requires overcoming some obstacles, including off-target effects and a lack of cell-specific targeting. Further efforts are needed to explore the therapeutic potential of this strategy.

## 5 Conclusions

This study proves that miR-542 inhibition therapy alleviates the severity of ICU-AW by inducing ATG5 to inhibit autophagy and mitigate mitochondrial damage. The potential of PEI/MMNs complexes as a non-viral polymer platform for safe and effective delivery of LNA to inhibit miRNA has been established. *In vivo* studies confirm that intraperitoneal injection of PEI/MMNs@LNA-542 NPs can suppress miR-542 expression and alleviate the severity of ICU-AW. This miRNA inhibitor delivery system provides a potential strategy for ICU-AW treatment.

## Supplementary Material

Supplementary Table
