# Supplementary material for: PEI/MMNs@LNA-542 nanoparticles alleviate ICU-acquired weakness through targeted autophagy inhibition and mitochondrial protection
Source: Open Life Sci. 2024 Sep 9;19(1):20220952. doi: 10.1515/biol-2022-0952 (PMC11406224; doi:10.1515/biol-2022-0952)
Supplement: Supplementary Table [file biol-2022-0952-sm.pdf]

Supplementary material

Table S1: Sequence of primer

Primer	Sequence
mmu-miR-542-5p	5′-CUCGGGGAUCAUCAUGUCACGA-3′
mmu-miR-542-3p	5′-UGUGACAGAUUGAUAACUGAAA-3′
hsa-miR-542-5p	5′-UCGGGGAUCAUCAUGUCACGAGA-3′
hsa-miR-542-3p	5′-UGUGACAGAUUGAUAACUGAAA-3′
U6	F-5′-CTCGCTTCGGCAGCACATATACT-3′
	R-5′-ACGCTTCACGAATTTGCGTGTC-3′
